# Optogenetic activation of the diaphragm

**DOI:** 10.1038/s41598-022-10240-w

**Published:** 2022-04-20

**Authors:** Ethan S. Benevides, Michael D. Sunshine, Sabhya Rana, David D. Fuller

**Affiliations:** 1 Rehabilitation Science PhD Program, University of Florida, Gainesville, Florida USA; 2 Department of Physical Therapy, University of Florida, Gainesville, Florida USA; 3 Breathing Research and Therapeutics Center, University of Florida, Gainesville, Florida USA; 4 McKnight Brain Institute, University of Florida, Gainesville, Florida USA

**Keywords:** Respiration, Optogenetics, Electromyography - EMG

## Abstract

Impaired diaphragm activation is common in many neuromuscular diseases. We hypothesized that expressing photoreceptors in diaphragm myofibers would enable light stimulation to evoke functional diaphragm activity, similar to endogenous bursts. In a mouse model, adeno-associated virus (AAV) encoding channelrhodopsin-2 (AAV9-CAG-ChR2-mVenus, 6.12 × 10^11^ vg dose) was delivered to the diaphragm using a minimally invasive method of microinjection to the intrapleural space. At 8–18 weeks following AAV injection, mice were anesthetized and studied during spontaneous breathing. We first showed that diaphragm electromyographic (EMG) potentials could be evoked with brief presentations of light, using a 473 nm high intensity LED. Evoked potential amplitude increased with intensity or duration of the light pulse. We next showed that in a paralyzed diaphragm, trains of light pulses evoked diaphragm EMG activity which resembled endogenous bursting, and this was sufficient to generate respiratory airflow. Light-evoked diaphragm EMG bursts showed no diminution after up to one hour of stimulation. Histological evaluation confirmed transgene expression in diaphragm myofibers. We conclude that intrapleural delivery of AAV9 can drive expression of ChR2 in the diaphragm and subsequent photostimulation can evoke graded compound diaphragm EMG activity similar to endogenous inspiratory bursting.

## Introduction

The emergence of optogenetic technology has provided a novel method for activating neurons with a high degree of temporal and spatial precision. With this approach, light activated ion channels are inserted into cell membranes as a result of genetic manipulation. Methods that are commonly used to express light activated ion channels include transgenic breeding schemes^1–3^ and delivery of opsin genes through viral vectors^4–6^. While most efforts have focused on neuronal activation, optogenetics can also be used to depolarize skeletal myofibers^7–9^ and mitigate limb muscle atrophy following denervation^10^.

The diaphragm separates the thoracic and abdominal cavities and is the primary muscle of inspiration^11^. Compared to other skeletal muscles, the diaphragm has: a high duty cycle, greater relative rate of blood flow, and a high susceptibility to disuse atrophy^12^. Diaphragm and respiratory dysfunction are also primary contributors to morbidity and mortality in many neuromuscular diseases^13,14^. Current approaches to combat respiratory dysfunction associated with diaphragmatic impairment include respiratory strength training^15,16^, diaphragm pacing using electrical stimulation^17–19^, and mechanical ventilation^20,21^. However, morbidity and mortality remain high in conditions associated with diaphragm dysfunction^22,23^.

In an effort to expand the possibilities for activating the diaphragm in neuromuscular disease, here we explored the use of optogenetic technology to stimulate recruitment of diaphragm motor units. To drive expression of a light sensitive protein in the murine diaphragm, an adeno-associated virus (AAV) encoding channelrhodopsin-2 (ChR2) was delivered via microinjection between the lung parietal pleural membrane and the visceral pleural membrane. This simple and minimally invasive AAV delivery method is translatable to human use^24^, and in our study effectively drove transgene (ChR2) expression in diaphragm myofibers. Using this approach, we tested the hypothesis that following ChR2 expression in diaphragm myofibers, directing appropriate light stimuli at the inferior diaphragm surface could produce graded diaphragm EMG activation, similar to the normally occurring endogenous inspiratory activation. To test this hypothesis, in an initial series of experiments, we systematically varied the intensity and duration of light stimuli directed at the diaphragm. After optimizing the light stimulus parameters, the functional impact of optogenetic diaphragm stimulation was evaluated by measuring respiratory airflow and diaphragm EMG activation in spontaneously breathing mice. This work included studies of a functionally impaired diaphragm to verify if the optogenetic stimulation could sustain breathing under such conditions.

## Materials and methods

### Experimental animals

All experiments were carried out using C57/bl6 mice (Taconic). An equal number of both sexes were injected with AAV at 6–7 weeks of age (n = 10 females; n = 10 males). Six animals were excluded due to equipment failure during electrophysiology experiments; a total of 14 animals were included in the final data analysis (n = 5 males; n = 9 females). Animals were housed five to a cage in a controlled environment (12 h light–dark cycle) with food and water ad libitum. All experiments were conducted in accordance with the NIH Guidelines Concerning the Care and Use of Laboratory Animals and were approved by the University of Florida Institutional Animal Care and Usage Committee. All work was done following the recommendations outlined in the ARRIVE guidelines.

### Intrapleural injections

Mice received a unilateral, intrapleural injection of AAV9-CAG-ChR2-mVenus (20071-AAV9 (Addgene), titer: 1.8 × 10^13^ vg/ml, dose: 6.12 × 10^11^ vg; 34 µl virus; 400 µl diluted) into the left intrapleural space. Animals were anesthetized under 2% isoflurane (in 100% O_2_) and placed on a heating pad kept at 37 °C to maintain body temperature (model 700 TC-1000, CWE). An insulin syringe (30-gauge needle) was used to inject 34 µl of AAV9- CAG-ChR2-mVenus diluted to 400 µl in sterile saline into the left intrapleural space via the fifth intercostal space. After the injection, isoflurane was turned off and mice were maintained on a heating pad until they righted themselves, at which point mice were returned to their home cage.

### Terminal electrophysiology

Animals underwent terminal electrophysiological recordings 8–18 weeks after intrapleural injection. Mice were anesthetized with 2% isoflurane and placed supine on a heating pad to maintain core body temperature at 37 ± 0.5 °C (model 700 TC-1000, CWE). To record diaphragm EMG activity, we performed a laparotomy consisting of a midline incision through the skin and abdominal muscles starting from the xiphoid process moving caudally down the linea alba. EMG activity of the mid-costal region of the left and right hemi-diaphragm was recorded using two pairs of 50 µm tungsten wires. Tips of the wires were de-insulated and formed into small hooks. The tips of the wires were inserted through the diaphragm approximately 3 mm apart on each side of the diaphragm. The recorded EMG signals were amplified (1000x) and filtered (100–1000 Hz) using a differential amplifier (A–M systems model 1700). Signals were digitized at 10 kS/s using a Power 1401 (CED, Cambridge, UK). After baseline recording periods, all animals underwent either a unilateral or bilateral phrenicotomy depending on experiment (see next section for details). Briefly, an incision was made on the ventral surface of the neck, the sternocleidomastoid was cut and reflected back. Just caudal to the sternocleidomastoid, the phrenic nerve was identified at the level of the brachial plexus and transected. Photostimulation of the diaphragm was achieved using a high-powered, 473 nm light emitting diode (LED, XQEBLU-00-0000-000000Z02, Cree Inc., Durham, North Carolina) controlled by the Power 1401 via an LED driver (PAM2861CBR, Diodes Incorporated, Plano, Texas). The abdominal incision was held open with alligator clips and the LED was directed through the laparotomy aimed at the inferior surface of the diaphragm. In a subset of animals, a tracheostomy tube (PE90 tubing) was placed to enable measurements of respiratory flow using a custom made pneumotachograph connected to a pressure transducer (SDP816-125PA, Sensirion, Stäfa, Switzerland).

### Experiment 1: characterization of diaphragm EMG response to photostimulation

To assess diaphragm EMG response to single pulses of light at varied light pulse intensities and durations, mice (n = 4; n = 1 female; n = 3 males; 15–18 weeks old) were anesthetized and EMG activity was recorded as described above. Animals in this experiment incubated for 8–9 weeks before undergoing diaphragm photostimulation.

In single pulse trials, we tested two stimulus response curves, one which varied light intensity and another which varied the duration of light pulses. The intensity trials consisted of 1 ms pulses of light that ranged from 10 to 60 mW/mm^2^. Light was delivered 10 times at each intensity to enable a stimulus triggered average of the evoked responses. Within the duration trials, the intensity of the light was held constant at 23 mW/mm^2^ and duration of the light pulses increased from 0.1 to 1 ms with 10 repeats at each duration. Both stimulus response curves were performed with both phrenic nerves intact and immediately following a right unilateral phrenicotomy. Diaphragm responses were quantified as peak-to-peak amplitude of the evoked response in the stimulus triggered average.

### Experiment 2: diaphragm response to light trains

Preliminary studies of how the diaphragm responded to several different trains of light pulses (see Table 1) were performed at the conclusion of experiment 1, after unilateral phrenicotomy. The light intensity in every train was ramped from 10 to 60 mW/mm^2^ over a 125 ms period, and then subsequently decreased from 60 to 10 mW/mm^2^, also over 125 ms. We also performed several stimulus response curves by delivering trains of light pulses with four different inter-pulse intervals (IPIs). The IPI for each stimulus response curve was held constant at the following four intervals: 0.1, 0.5, 1, and 5 ms. Within each curve the pulse duration (PD) was varied from 0.1 to 1.0 ms in 0.1 ms steps with 5 repeats at each duration. The stimulus reached a maximum of a 50% duty cycle (e.g. 0.1 ms pulse duration and 0.1 ms inter-pulse interval). These stimulation paradigms included inter-stimulus intervals that are faster than the ChR2 closing kinetics (~ 20 ms)^25^. Therefore, the repeated stimuli in trains with inter-stimulus intervals < 20 ms may not have excited ChR2 channels. These fast trains were used to mimic the effects of continuous light, but with stimulus parameters that mitigated heat from the light source. From these data, we identified four stimulus paradigms of interest that were explored further in additional experiments.

**Table 1:**
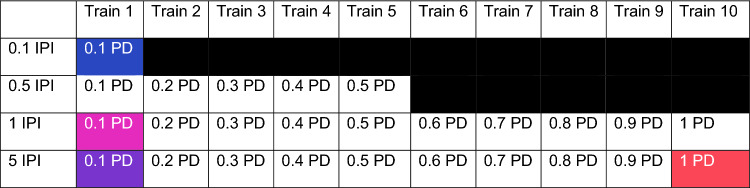
Parameters of light pulse trains.

The inter-pulse intervals (IPI) and pulse durations (PD) used to stimulate the diaphragm were systematically varied across ten stimulus trains. Parameters were not used if they exceeded a 50% duty cycle (black boxes). The colored boxes indicate the IPI/PD combinations that were selected for comprehensive testing in experiment two.

In a second cohort of mice (n = 5; n = 4 females; n = 1 male; 20–21 weeks old) we validated how the diaphragm responded to these stimulus trains following bilateral phrenicotomy. Animals in this experiment incubated for 9–12 weeks before undergoing diaphragm photostimulation. The stimulus parameters were chosen based on preliminary experiments, as follows. The IPI 5 ms/PD 1 ms paradigm evoked the largest diaphragm EMG bursts and was therefore selected for further testing. The evoked bursts within the other inter-pulse intervals did not increase as pulse duration increased, so we selected the shortest pulse duration to minimize heating of the LED. The four patterns of stimulation that were chosen are highlighted in Table 1.

To enable a subsequent contrast between the light-evoked activity and endogenous EMG activity, diaphragm EMG activity was first recorded for 10 minutes during spontaneous breathing (baseline). Following the baseline recording, the left and right phrenic nerves were transected near the brachial plexus. Diaphragm photostimulation was initiated immediately following the bilateral phrenicotomy. The inter-train interval was set equal to the expiratory duration that was recorded in each animal during baseline. This enabled the light-evoked diaphragm bursts to occur at the same rate as spontaneous breathing. In these experiments, animals underwent diaphragm photostimulation with each of the four-stimulus trains. The stimulus paradigms were presented in random order and each animal underwent one minute of photostimulation per paradigm for a total of five minutes of stimulation.

### Experiment 3: assessment of evoked diaphragm responses over a 1 hour photostimulation period

The purpose of this experiment was to determine if diaphragm responses to repeated photostimulation were stable over a 1 hour period. Mice (n = 5; n = 4 females; n = 1 male; 28–30 weeks old) were anesthetized and EMG recordings made as described above. Animals in this experiment incubated for 17–18 weeks before undergoing diaphragm photostimulation. After a 10 minute baseline recording the right phrenic nerve was transected. To ensure that photostimulation did not interfere with endogenous respiratory activity, it delivered in a “closed loop” fashion, using the EMG activity of the intact hemi-diaphragm as a stimulus trigger. The stimulation consisted of 250 ms trains that ramped from 10 to 60 mW/mm^2^ and then decremented back to 10 mW/mm^2^. The stimulus trains used a 0.1 ms pulse duration and 0.1 ms inter-pulse interval.

### Data analysis

A custom MATLAB (MathWorks, Natick, Massachusetts) script was used to compile stimulus triggered averages and cycle triggered averages of diaphragm EMG and respiratory airflow data. Per prior reports^26^ we formed stimulus triggered averages of the single pulse data using a window that began 5 ms before the onset of stimulation and ended 15 ms after the onset of stimulation. This stimulus triggered average was then used to calculate the peak to peak amplitude of the evoked response. EMG data are presented in normalized units as well as absolute units (mV). Normalization of light-evoked EMG responses was done as follows. For experiments in which the stimulus intensity was varied, the EMG response was expressed relative to the response which occurred at the maximum stimulus. For experiments in which the stimulus duration was varied, the evoked response was expressed relative to the EMG at the longest stimulus duration. For experiments where trains of pulses were used the EMG activity was rectified and integrated (50 ms time constant). To fully capture the longer latency responses, the window size was adjusted to 150 ms before the onset of stimulation and ended 450 ms after the onset of stimulation. To compare light-evoked diaphragm activity with endogenous bursting, cycle-triggered averages were calculated using a window size equal to the duration of the endogenous EMG burst^27^. The stimulus and cycle-triggered averages were used to calculate peak amplitude and area under the curve. To determine the power in the EMG bursts, the average power in frequency bands from 0 to 1001 Hz was calculated in 333 Hz increments. Additionally, a fast Fourier transform of diaphragm EMG bursts was done during baseline, after bilateral phrenicotomy, and during the different stimulus conditions. Statistical analyses were performed in SigmaPlot (Systat Software Inc., San Jose, California). Tests of normality and equal variance were performed for each data set. Non-parametric tests were performed for data sets that failed tests of equal variance. Square root transformation was used to transform data that failed the test of normality. The statistical tests used for each dataset are listed in the figure legends. Results of statistical tests were evaluated with an a priori alpha value of 0.05.

### Histology

At the conclusion of the electrophysiology experiments, while mice remained under isoflurane anesthesia, the diaphragm was rapidly removed and mice were then euthanized via transcardial perfusion. Hemi-diaphragm strips were laid flat and rolled into a spiral, rolling from the dorsal most edge of the diaphragm to the ventral edge. This ensured that each cross section would include representative fibers across the dorsal–ventral axis. The rolled diaphragms were then frozen in optimal cutting temperature media (OCT) and stored at − 80 °C until sectioning. Frozen diaphragms were cryo-sectioned (20 µm thickness) onto super-frost plus slides and stained with wheat germ agglutinin conjugated to Alexa Fluorophore 647 (1:250 dilution, 120 min incubation) to identify the membranes of the muscle fibers. Tissue was imaged with a fluorescence microscope (BZ-X710, Keyence Co., Osaka, Japan) using a 10 × objective. To visualize the mVenus and Alexa-Fluorophore 647 fluorescence, images were captured using GFP and Cy5 cube respectively. Four tissue sections from the mid costal diaphragm (spaced 80 µm apart) were imaged from each animal. The costal diaphragm was evaluated because it makes a primary contribution to the inspiratory effort^28^. Myofiber transduction and cross sectional area were assessed using a MATLAB script. The script identified the myofiber boundaries using the image captured with the Cy5 cube.

To determine the number of mVenus positive myofibers in each section, the intensity of mVenus fluorescence within each identified myofiber was determined. Myofibers were considered to be positive for mVenus if the intensity of fluorescence was above a threshold (median intensity + 1.96 median absolute deviations). This threshold was determined through the use of a histogram of the image pixel intensity. Prior work has established histogram based thresholding as an accurate method of automatic image segmentation^29^. Typically, histograms of image intensity produce two or more peaks that represent background objects and objects of interest. A threshold to separate the peaks is then determined by calculating the mean and standard deviation of pixel intensity. In a heavily skewed histogram, the median more accurately describes the center of the data and the median absolute deviation can be used to estimate variance^29^. Here the histogram of cell intensities was positively skewed and thus we based our threshold on the median and median absolute deviation of the data set. The percent transduction for each section was determined by counting the number of mVenus positive myofibers and expressing that value relative to the total number of diaphragm myofibers. Myofiber cross-sectional area was calculated by summing the total number of pixels within the boundaries of each detected myofiber and converting pixels to µm^2^ using a conversion factor obtained by determining the number of pixels across a known distance.

### Ethical declarations

All experiments were conducted in accordance with the NIH Guidelines Concerning the Care and Use of Laboratory Animals and were approved by the University of Florida Institutional Animal Care and Usage Committee. All work was done following the recommendations outlined in the ARRIVE guidelines.

## Results

### Increasing stimulus intensity and duration increases magnitude of diaphragm EMG response

**Figure 1:**
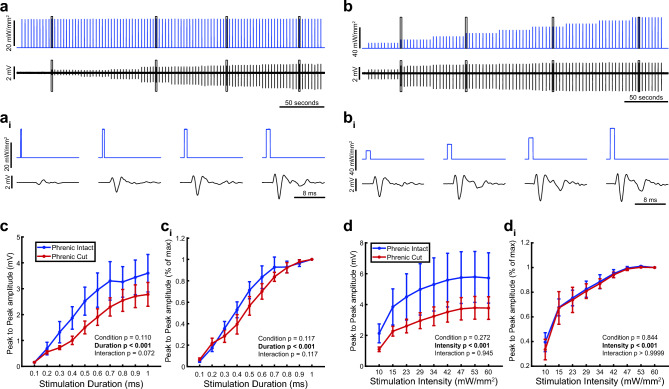
Diaphragm responses to single pulse photostimulation. The top panels show representative data traces from experiments in which pulse duration (**a**) or light intensity (**b**) were systematically varied. The expanded time scale traces shown in panels (**a**_**i**_) and (**b**_**i**_) allow viewing of evoked diaphragm EMG potentials. The average peak-to-peak amplitude of diaphragm evoked potentials in response to increasing pulse duration are shown in (**c**); average responses to increasing light intensity are shown in (**d**) (n = 4 per group). In both experiments, photostimulation was done before (blue) and after (red) unilateral phrenicotomy. Light stimulation was directed at the hemi-diaphragm ipsilateral to the phrenicotomy. Data are presented in absolute units (mV, panels **c** and **d**) or normalized to the peak to peak amplitude response at maximum stimulus intensity (panels **c**_**i**_ and **d**_**i**_). Panel (**c)**: 2-way RM ANOVA showed a significant effect of pulse duration (*p* < 0.001). The trend for reduced amplitude after phrenicotomy was not statistically significant (condition *p* = 0.110), nor was the interaction between duration and condition (*p* = 0.072). Panel (**c**_**i**_): 2-way RM ANOVA (square root transformed, non-transformed data plotted), significant effect of duration (*p* < 0.001) but not condition (*p* = 0.117) or interaction (*p* = 0.117). Panel (**d**): 2-way RM ANOVA: significant effect of duration (*p* < 0.001) and no effect of condition (*p* = 0.272) or interaction (*p* = 0.945). Panel (**d**_**i**_): 2-way RM ANOVA, significant effect of intensity (*p* < 0.001) but not condition (*p* = 0.844) or interaction (*p* > 0.9999).

The first experiments evaluated how increasing the duration of light pulses impacted the evoked diaphragm EMG responses. The intensity of the light stimulus was maintained at 23 mW/mm^2^ and the duration of pulses was varied from 0.1 to 1 ms (example shown in Fig. 1a-a_i_). We also assessed the effect of intensity by holding the stimulus duration constant at 1 ms while varying the light intensity from 10 to 60 mW/mm^2^ (example shown in Fig. 1b-b_i_). The first experiments showed that increasing the pulse duration caused a progressive increase in the evoked EMG response. The EMG responses are shown in absolute units (i.e., mV, Fig. 1c) or normalized to the peak response (i.e., %maximum, Fig. 1ci). These experiments were repeated after a unilateral section of the phrenic nerve (i.e., phrenicotomy). As shown in Fig. 1c,d, after phrenicotomy the diaphragm response still showed a progressive increase in parallel with the pulse duration, but there was suggestion of an attenuated response. The second series of experiments showed that the amplitude of the evoked diaphragm response increased with the intensity of the light pulse (*p* < 0.001, Fig. 1d). The raw diaphragm EMG (mV) showed considerable variability between experiments, but this was largely removed by the normalization to the maximum response (Fig. 1di). As the light intensity was increased, the diaphragm EMG response plateaued around 47 mW/mm^2^ intensity. As with the duration experiments, the unilateral phrenicotomy tended to reduce the amplitude of the evoked diaphragm EMG potential (Fig. 1d *p*  =  0.272).

### Photostimulation can evoke diaphragm EMG bursts that are similar to inspiratory EMG activity

**Figure 2:**
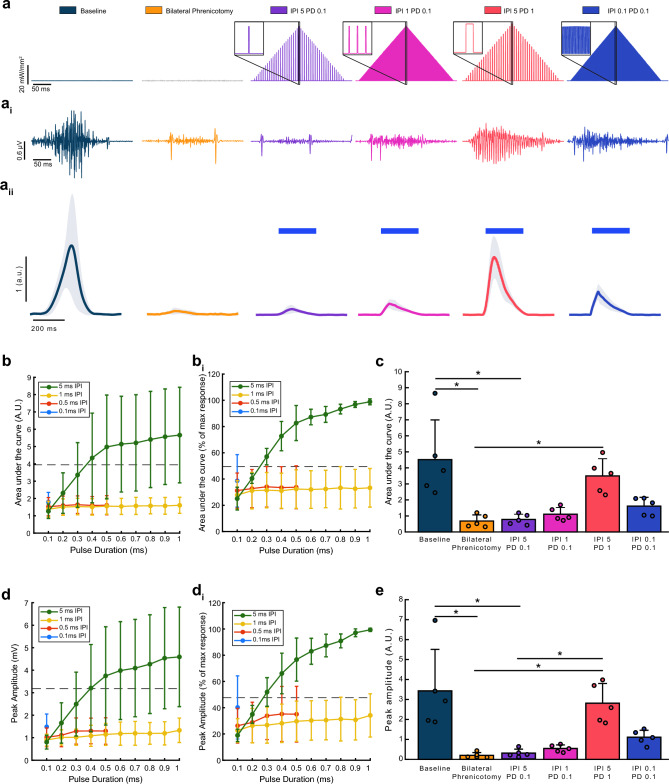
Impact of light train inter-pulse interval and pulse duration on the evoked diaphragm EMG response. (**a**) Graphical depiction of the four different stimulus trains. (**a**_**i**_) Representative EMG responses are shown beneath each paradigm. (**a**_**ii**_) Rectified and integrated diaphragm EMG activity averaged across animals (n = 5). Depicted are cycle triggered averages of inspiratory EMG bursting during spontaneous breathing at baseline and following bilateral phrenicotomy. Also depicted are stimulus triggered averages for light-evoked EMG activation. The solid color lines represent mean data and the shaded region represents one standard deviation. The blue bars represent onset and duration of stimulation. (**b**) Diaphragm EMG responses, presented as area under the curve of integrated EMG bursts, (n = 4, unilateral phrenicotomy) to 250 ms trains of light pulses. Pulse durations ranged from 0.1 to 1 ms and inter-pulse intervals of 0.1, 0.5, 1, and 5 ms. (**b**_**i**_) Area under the curve analysis, presented as percentage of maximal evoked response. Grey dotted lines represent average area under the curve during endogenous diaphragm bursting. (**c**) Area under the curve for the integrated diaphragm EMG bursts (n = 5, bilateral phrenicotomy), for each of the four stimulation paradigms tested (one-way repeated measures ANOVA on ranks, *p* < 0.001, *Tukey’s post-hoc comparison *p* < 0.05, horizontal lines indicate significant post-hoc differences between the two histograms where the line starts and ends). Data are presented as the average area under the curve + 1 stdev. (**d**) Peak diaphragm EMG amplitude response (n = 4, unilateral phrenicotomy) to short trains of light pulses with pulse durations ranging from 0.1 to 1 ms and inter-pulse intervals of 0.1, 0.5, 1, and 5 ms. (**d**_**i**_) Peak amplitude diaphragm EMG response, as a percentage of maximal evoked response, to short trains of light pulses with pulse durations ranging from 0.1 to 1 ms and inter-pulse intervals of 0.1, 0.5, 1, and 5 ms. Grey dotted lines represent average peak amplitude during endogenous diaphragm bursting. (**e**) Peak amplitude (n = 5, bilateral phrenicotomy) for stimulation paradigms of interest (one-way repeated measures ANOVA on ranks *p* < 0.001, *Tukey’s post-hoc comparison *p* < 0.05, horizontal lines indicate significant post-hoc differences between the two histograms where the line starts and ends). Data are presented as the average peak amplitude + 1 stdev.

We next used trains of light stimuli in an effort to produce diaphragm EMG bursting which resembled the endogenous inspiratory burst (Fig. 2a). A range of stimulus–response curves were performed after phrenicotomy to determine which stimulus parameters produced bursting activity similar to the inspiratory burst. The inter-pulse interval was held at 0.1, 0.5, 1, or 5 ms, and at each interval the duration of light pulses was progressively ramped from 0.1 to 1 ms (0.1 ms increments). Representative evoked diaphragm responses are shown in Fig. 2ai, and stimulus triggered averages showing the integrated diaphragm EMG responses are shown in Fig. 2aii. These data illustrate that the 5 ms inter-pulse interval trains produced the largest EMG response.

Unilateral phrenicotomy reduced endogenous excitatory synaptic inputs to the diaphragm, and allowed us to determine if inspiratory-like activity could be restored by the light stimulation. The 5 ms IPI increased EMG area under the curve after unilateral (Fig. 2b-bi) and bilateral (Fig. 2c) phrenicotomy. This trend held for peak amplitude (Fig. 2d–e) as pulse duration increased. Stimulus trains with 0.1, 0.5, and 1 ms inter-pulse intervals, regardless of pulse duration, evoked similar diaphragm EMG responses (Fig. 2b-bi, d-di). Based on these results, we identified four inter-pulse interval/ pulse duration combinations (5 ms IPI/ 0.1 ms PD, 1 ms IPI/0.1 ms PD, 5 ms IPI/1 ms PD, and 0.1 ms IPI/0.1 ms PD) to investigate over a longer, one minute, time period in a bilateral phrenicotomy model (Fig. 2c,e).

Additional experiments were conducted following bilateral phrenicotomy. This allowed us to determine if photostimulation could evoke inspiratory-like diaphragm bursting under conditions of severe diaphragm impairment. The bilateral phrenicotomy reduced, but did not completely eliminate, EMG activity recorded via the diaphragm electrodes (Supplemental Fig. 1). It is likely that the residual diaphragm EMG activity occurred due to synaptic inputs provided by the accessory phrenic nerve^30^. The accessory phrenic nerve is a small nerve that originates from C6 and joins the common phrenic nerve in the thorax at various locations, making it difficult to identify^31^. Since we targeted the common phrenic for the transection, the residual EMG activity was likely due to the accessory nerve. Overall, stimulation with trains of light pulses, evoked robust compound diaphragm EMG activity after bilateral phrenicotomy.

The stimulus paradigms with short pulse durations and long inter-pulse durations (5 ms IPI/0.1 ms PD, 1 ms IPI/0.1 ms PD) evoked small EMG bursts that were similar in magnitude to the diaphragm activity after phrenicotomy (Fig. 2a-a_ii_). The 0.1 ms IPI/0.1 ms PD train evoked dense, compound bursts that were not statistically different in magnitude from any of the other tested paradigms (Fig. 2c,e). The 5 ms IPI/1 ms PD train evoked the largest EMG output, however, the evoked burst resembled repeated compound twitches and did not have the density associated with endogenous bursts (Fig. 2ai).

Power spectral analysis revealed the 5 ms IPI/1 ms PD train paradigm evoked an EMG response with high power in a narrow, low frequency band (180–190 Hz) which likely explains the saw tooth appearance of the evoked bursts (Supplemental Fig. 2). To ensure the observed response was due to photostimulation of ChR2 and not light alone, the same LED was shone onto the diaphragm of mice that did not receive an intrapleural injection of AAV after unilateral phrenicotomy (Supplemental Fig. 3). Shining light on the diaphragm of mice that had not been transduced with AAV produced no discernable EMG response.

**Figure 3:**
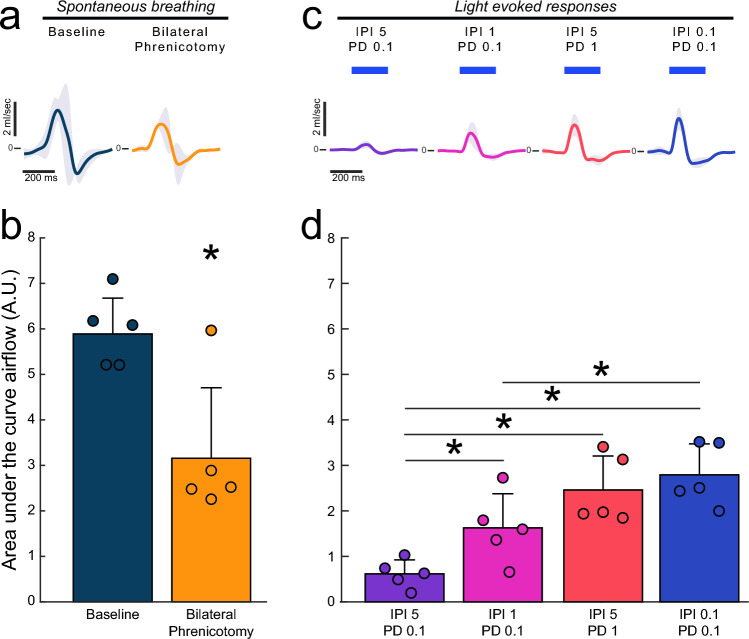
Diaphragm photostimulation can produce respiratory airflow. (**a**) Cycle triggered averages of airflow across the respiratory cycle during spontaneous breathing at baseline and immediately after the bilateral phrenicotomy. The upward deflection represents inspiration. Solid color lines represent mean data, shaded regions represent one standard deviation. The average respiratory airflow under these conditions is shown in panel (**b**). Paired t-tests showed a difference in airflow between baseline and bilateral phrenicotomy (*p* = 0.045). (**c**) Stimulus triggered averages of the respiratory flow waveform across all animals (n = 5). Blue bars represent onset and duration of stimulation. Panel (**d**) presents the area under the curve of the evoked airflow traces for the four stimulus conditions tested. Data are presented as mean + 1 stdev with scatter showing data for individual animals. One way repeated measures ANOVA showed an effect of stimulation on airflow (*p* < 0.001), black lines indicated significant differences between groups (*p* < 0.05).

A pneumotachograph was used to evaluate the impact of diaphragm EMG bursting on respiratory airflow. The bilateral phrenicotomy produced a reduction of inspiratory airflow in 4 of 5 mice (Fig. 3a–b). One mouse did not show a reduction in airflow, presumably due to rapid compensatory recruitment of accessory respiratory muscles. After phrenicotomy, the impact of light-induced diaphragm contraction on airflow was evaluated (Fig. 3c–d). Importantly, this was done during the expiratory phase of the respiratory cycle so that the impact of light activation could be evaluated without contraction of accessory inspiratory muscles. The 5 ms IPI/0.1 ms PD stimulus train, which produced the smallest evoked EMG response, generated the least airflow (Fig. 3c–d). The 1 ms IPI/0.1 ms PD train evoked a larger magnitude airflow (*p* < 0.001 vs. the 5 ms IPI/0.1 ms PD train, Fig. 3d). The two paradigms that evoked the largest EMG responses also generated the most airflow (Fig. 3c–d). The 5 ms IPI/1 ms PD train evoked airflow that was significantly greater than the 5 ms IPI/0.1 ms PD train and the 1 ms IPI/0.1 ms PD trains (p < 0.001). Similarly, the 0.1 ms IPI/0.1 ms PD train generated airflow that was significantly greater than both the 5 ms IPI/0.1 ms PD and 1 ms IPI/0.1 ms PD stimulus trains (*p* < 0.001, Fig. 3d).

### Photostimulation can pace the diaphragm over a prolonged period without a decrease in evoked response magnitude

**Figure 4:**
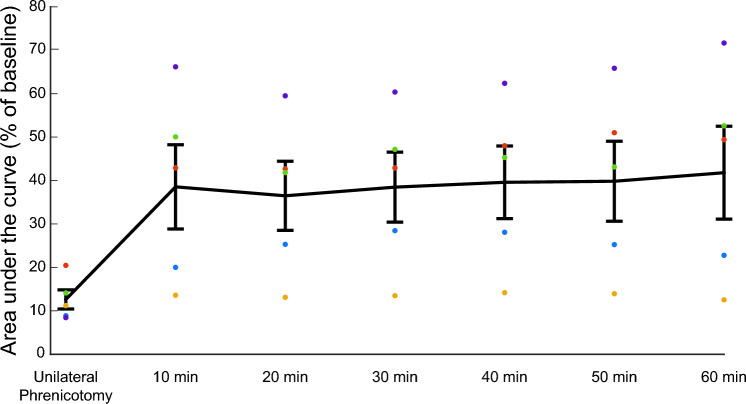
Light-induced diaphragm EMG activation is not attenuated over 1 hour of stimulation. Mice received a unilateral phrenicotomy and then closed loop photostimulation of the paralyzed hemi-diaphragm in phase with respiration using a 250 ms train of light pulses with inter-pulse interval of 0.1 ms and light pulse duration of 0.1 ms. Data are presented as a percent of baseline. Black line represents the mean + /− stdev. Individual scatter points represent each animal. After unilateral phrenicotomy there is a pronounced attenuation in EMG output. Photostimulation increases diaphragm EMG output of the paralyzed hemi-diaphragm. Ten-minute averages of evoked EMG activity show no attenuation of evoked bursts over a sixty-minute period of closed-loop, inspiratory triggered diaphragm photostimulation. Friedman Repeated Measures Analysis of Variance on Ranks showed a significant effect of time on evoked response (*p* = 0.017).

To assess the effect of prolonged stimulation on the magnitude of evoked diaphragm EMG responses, we performed an experiment where mice received a unilateral phrenicotomy followed by photostimulation of the hemi-diaphragm ipsilateral to the phrenicotomy. Stimulation was delivered in a closed-loop fashion with endogenous activity on the intact hemi-diaphragm triggering trains of light pulses. Thus, the onset of the inspiratory burst of EMG activity on the intact diaphragm served as the trigger for stimulating the paretic diaphragm. Unilateral phrenicotomy resulted in attenuation of ipsilateral hemi-diaphragm activity. When stimulation began there was a subsequent increase in the magnitude of the EMG area under the curve (Fig. 4). Following the initial increase in burst magnitude, the values stayed consistent over a sixty-minute period of stimulation (Fig. 4).

### Transduction of diaphragm myofibers

**Figure 5:**
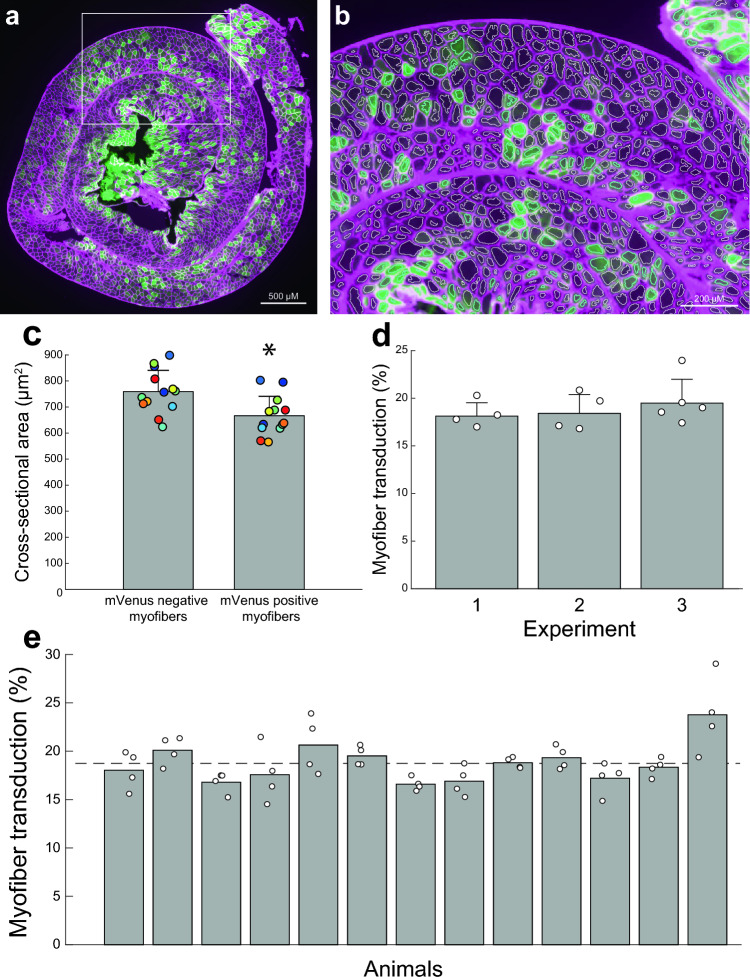
Transduction of diaphragm myofibers following intrapleural microinjection of AAV9. (**a**) Representative diaphragm histology. The diaphragm was rolled prior to sectioning so that cross sections enabled visualization of fibers throughout the costal region. The green myofibers show the presence of the mVenus fluorescence tag. (**b**) Inset from panel (**a**), myofibers identified by the MATLAB analysis script are outlined with white borders, mVenus positive fibers can be appreciated by the green color and are outlined with blue borders. (**c**) Diaphragm myofiber cross-section area for mVenus positive and mVenus negative myofibers. The scatter plot shows the average cross-sectional area for mVenus positive and negative myofibers from each animal (n = 13). Color of scatter corresponds to each individual animal. Wilcoxon rank sum test suggests a significant difference between cross-sectional area of mVenus positive and negative myofibers (*p* = 0.008). (**d**) Comparison of diaphragm transduction across the three different experiments. One way ANOVA suggested no differences in percent transduction between experiments (*p* = 0.59). (**e**) Percent transduction for each individual animals (n = 13). The scatter plot shows the data for each individual tissue section (n = 4 per mouse). The histogram shows the average for each mouse and the dotted line shows average percent transduction across all animals.

The costal diaphragm contained clusters of mVenus positive myofibers separated by clusters of myofibers with no detectable mVenus (Fig. 5a–b). The cross-sectional area of the mVenus negative myofibers (759 ± 82 µm^2^) was larger than the mVenus positive myofibers (666 ± 75 µm^2^) (Fig. 5c *p* = 0.008). The relative transduction of diaphragm myofibers was comparable across the three different experimental paradigms (Fig. 5d *p* = 0.5). Across all experiments, an average of 18 ± 2% of diaphragm myofibers expressed mVenus (range of 17–24%; Fig. 5e). The number of detectable diaphragm myofibers expressing mVenus stayed consistent across the 8–18 week AAV incubation period with values averaging 18% ± 2% after 8 weeks, and 19% ± 3% after 18 weeks.

## Discussion

Respiratory insufficiency contributes to morbidity and mortality in individuals with neuromuscular disease^14^ and spinal cord injury^13^. As the primary inspiratory muscle, the diaphragm is an ideal target for therapeutic interventions to preserve respiratory functioning. Here we have opened up a new avenue of exploration for targeted diaphragm activation via optogenetic technology. We demonstrate that an intrapleural microinjection of AAV9 encoding ChR2 can effectively drive ChR2 expression in diaphragm myofibers. Subsequent photostimulation can activate diaphragm myofibers, and light stimulus trains produce functionally effective EMG bursts similar to endogenous diaphragm muscle activity that occurs during inspiration.

To date, optogenetic technology has primarily been used to elucidate the function of neural circuits^32^. One prior study used optogenetics to activate cervical spinal neurons, which in turn activated the diaphragm. A ChR2-GFP Sindbis virus was injected into the mid-cervical spinal cord, and this produced a non-specific transduction of motoneurons and interneurons. Subsequently, light stimulation of the C3-C6 spinal cord was able to evoke diaphragm EMG bursting activity, even following cervical spinal cord injury^33^. Only a few prior studies have explored optogenetics as a tool for activating muscle cells, beginning with a study of cultured myocytes expressing ChR2^7^. Subsequent in vivo experiments have demonstrated light-activation of skeletal muscles which express ChR2^8,9^. For example, single light pulses can evoke skeletal (soleus) muscle twitches, and increasing stimulus intensity and duration increases the evoked force^9^. Our data confirmed a similar response in the diaphragm, with progressively larger EMG activation occurring with increases in light stimulus intensity and duration. A few prior reports showed that optogenetic techniques can also be effective in denervated skeletal muscle^10,34^, and here we observed that photostimulation could activate the paralyzed diaphragm even after acute section of the phrenic nerve. Thus, the paralyzed diaphragm could be induced to contract, although the EMG response to light stimulation was reduced compared to the phrenic nerve intact responses. The underlying mechanism for the reduction in evoked diaphragm activation after acute denervation is not clear, and the time course is too rapid (i.e., immediate reduction) to reflect degeneration of neuronal inputs. However, the result suggests a decrease in the excitability of diaphragm myofibers may have occurred immediately after the acute phrenicotomy.

Here we elected to use the relatively simple and minimally invasive method of microinjection to the pleural space to deliver AAV to the diaphragm. This method targets the “potential space” between the visceral pleura that lines the lungs and the parietal pleura which covers the inner surface of the thoracic cavity. Intrapleural viral delivery has previously been used to target pleural tumors^35,36^, and to drive gene expression in the diaphragm, lungs, and heart^37,38^. For example, after intrapleural injection of AAV9 (1 × 10^11^ vg) in a mouse model, AAV was detected in cardiac, diaphragm, and cervical spinal cord tissue^39^. Distribution in tissues other than the lung and diaphragm will likely always occur after intrapleural delivery because the pleural space communicates with the lymphatics^40^. Thus intrapleural delivery of viral vectors will likely always produce some degree of off-target gene expression. When planning for the current study, we conducted preliminary experiments in which we histologically assessed the mid-cervical spinal cord four months after mice received intrapleural AAV9-CAG-ChR2-mVenus. We were able to find no evidence of spinal cord expression of mVenus, although that possibility needs to be rigorously evaluated. Nevertheless, in the current work, the light source was focused directly on the diaphragm during photostimulation making it highly unlikely that activation of non-diaphragmatic cells potentially transduced by intrapleural AAV9 occurred.

In our study, intrapleural delivery of AAV9-CAG-ChR2-mVenus (dose: 6.12 × 10^11^ vg) produced gene expression in approximately 20% of diaphragm myofibers (as determined by evaluating diaphragm mVenus expression). Our transduction efficiency was similar to reported transduction efficiency for rodent intramuscular diaphragm AAV (24%)^41^. De et al. also reported robust transduction of diaphragm myofibers after intrapleural AAV5 injection, although the efficiency was not quantified^38^. There are several strategies for potentially increasing the relative number of diaphragm myofibers expressing the transgene. Here we used intrapleural delivery due to the non-invasive nature of this method^42^, but different AAV delivery strategies could improve diaphragm myofiber transduction. For example, more direct delivery of the AAV to the diaphragm, either by direct intramuscular injection^41^ or application of an AAV-containing gel medium^43,44^, could potentially increase myofiber transduction rates. A caveat, however, is that directly accessing the diaphragm requires considerably more invasive surgical approaches. Additionally, selecting a different AAV serotype could potentially improve diaphragm transduction efficacy^45^. An AAV serotype can be thought of as a combination of variations in amino acid residues that are exposed on the surface of the AAV capsid leading to variations in capsid structure and cell surface interactions^46^. Historically, AAV9 and AAV1 have shown excellent tropism for skeletal muscle^45^. Here we tested the AAV9 capsid, however, it may be worth testing an AAV1 vector which has produced robust diaphragm transduction in prior work^43^. In this proof-of-concept study, we did not evaluate possible immune responses to the AAV9 vector or transgene products. Immune responses which reduce the efficacy of diaphragm transgene expression are possible, but, responses in other skeletal muscles after AAV treatment suggests minimal immune response to AAV9 in rats and mice^47^. Lastly, additional factors that can impact the rates of AAV transduction include sex^48,49^ and age^50,51^. However, in the current study, diaphragm transduction rates were highly consistent across male and female animals, making it unlikely that sex differences were a factor. This discussion highlights the potential ways to increase diaphragm transduction; however, we emphasize that the intrapleural delivery method was sufficient to enable a distinct and sustainable diaphragm EMG response to light activation.

During quiet breathing, which can be considered a “low force” effort, approximately 20% of total diaphragm myofibers are active^52^. This number is similar to our estimation that approximately 20% of costal diaphragm myofibers were expressing the ChR2 transgene in the present study. Thus, our observation that light-activation of diaphragm myofibers is sufficient to produce diaphragm EMG activation patterns that resemble “normal” resting breathing is consistent with models of diaphragm motor unit recruitment. One caveat, however, is that we did not determine the specific diaphragm fiber types expressing the transgene. The diaphragm is a mixed muscle containing a full complement of slow twitch (Type I) and fast twitch (Type IIa, IIx, and IIb) fiber types. Quiet breathing requires primarily Type I diaphragm myofibers, and high force “expulsive” diaphragm behaviors such as coughing or vomiting require full recruitment of all available myofibers^52^. Thus, using ChR2 to enable higher force diaphragm behaviors is likely to require transduction of all myofiber types. A few studies have reported that AAV9 preferentially transduces slow twitch fibers^50,53^. Conversely, other reports indicate that AAV9 has a preference for fast twitch fibers^54,55^. Another study found no difference in AAV-mediated transduction across fiber type^56^. In the current study, the diaphragm muscle fibers expressing transgene-driven fluorescence had a smaller cross sectional area as compared to fibers that were not transduced. This result may indicate that Type I myofibers, which are smaller, had greater AAV transduction as suggested by a few prior reports^50,53^.

In conclusion, our study provides the first evidence that optogenetic technology can be used to activate the diaphragm in a manner that resembles endogenous inspiratory activity. These results open up new avenues for therapeutic targeting of diaphragm paresis or paralysis. Currently, resistive breathing exercises can be used to improve diaphragm function when volitional breathing remains possible^57^. When independent breathing cannot be sustained, clinical interventions to address diaphragm dysfunction include mechanical ventilation^58^, and direct diaphragm pacing using electrical stimulation^18,19^. This optogenetic technique could have implications for certain clinical populations, such as those with cervical spinal cord injury that includes damage to the phrenic nerve or phrenic motor pool. A common exclusion criteria for phrenic nerve stimulation is damage to the phrenic nerve and/or the phrenic motor pool^18,59^. This means that many individuals that could benefit from phrenic nerve stimulation, such as those with a cervical spinal cord injury, are ultimately deemed ineligible. Unlike traditional diaphragm/phrenic nerve pacing paradigms, the optogenetic stimulation technique proposed here works by directly activating diaphragm myofibers. Therefore, the technique has potential use in cases where individuals would otherwise by ineligible for electrical diaphragm/phrenic nerve pacing. Collectively, the current data serve as a proof-of-concept that expression of ChR2 in diaphragm myofibers permits rhythmic activation of this essential inspiratory muscle using a light stimulus.

## Supplementary Information

Supplementary Figure 1.

Supplementary Figure 2.

Supplementary Figure 3.

Supplementary Figure legends.

## Data Availability

The datasets and MATLAB code generated during the current study are available from the corresponding author on reasonable request.

## References

[1] Cohen JA (2019). Cutaneous TRPV1 neurons trigger protective innate type 17 anticipatory immunity. Cell.

[2] Zeng H, Madisen L (2012). Mouse transgenic approaches in optogenetics. Prog. Brain Res..

[3] Hibberd TJ (2018). Optogenetic induction of colonic motility in mice. Gastroenterology.

[4] Boyden ES, Zhang F, Bamberg E, Nagel G, Deisseroth K (2005). Millisecond-timescale, genetically targeted optical control of neural activity. Nat. Neurosci..

[5] Mondello SE (2018). Optogenetic surface stimulation of the rat cervical spinal cord. J. Neurophysiol..

[6] Mondello SE (2021). A micro-LED implant and technique for optogenetic stimulation of the rat spinal cord. Exp. Neurol..

[7] Asano T, Ishizua T, Yawo H (2012). Optically controlled contraction of photosensitive skeletal muscle cells. Biotechnol. Bioeng..

[8] van Bremen T, Send T, Sasse P, Bruegmann T (2017). Spot light on skeletal muscles: Optogenetic stimulation to understand and restore skeletal muscle function. J. Muscle Res. Cell Motil..

[9] Bruegmann T (2015). Optogenetic control of contractile function in skeletal muscle. Nat. Commun..

[10] Magown P, Shettar B, Zhang Y, Rafuse VF (2015). Direct optical activation of skeletal muscle fibres efficiently controls muscle contraction and attenuates denervation atrophy. Nat. Commun..

[11] Poole DC, Sexton WL, Farkas GA, Powers SK, Reid MB (1997). Diaphragm structure and function in health and disease. Med. Sci. Sports Exerc..

[12] Powers SK, Smuder AJ, Fuller D, Levine S (2013). CrossTalk proposal: Mechanical ventilation-induced diaphragm atrophy is primarily due to inactivity. J. Physiol..

[13] Berlowitz DJ, Wadsworth B, Ross J (2016). Respiratory problems and management in people with spinal cord injury. Breathe (Sheff).

[14] Bye PT, Ellis ER, Issa FG, Donnelly PM, Sullivan CE (1990). Respiratory failure and sleep in neuromuscular disease. Thorax.

[15] Ferreira GD, Costa AC, Plentz RD, Coronel CC, Sbruzzi G (2016). Respiratory training improved ventilatory function and respiratory muscle strength in patients with multiple sclerosis and lateral amyotrophic sclerosis: Systematic review and meta-analysis. Physiotherapy.

[16] Bissett BM, Leditschke IA, Neeman T, Boots R, Paratz J (2016). Inspiratory muscle training to enhance recovery from mechanical ventilation: A randomised trial. Thorax.

[17] Onders RP, Ponsky TA, Elmo M, Lidsky K, Barksdale E (2011). First reported experience with intramuscular diaphragm pacing in replacing positive pressure mechanical ventilators in children. J. Pediatr. Surg..

[18] Posluszny JA (2014). Multicenter review of diaphragm pacing in spinal cord injury: Successful not only in weaning from ventilators but also in bridging to independent respiration. J Trauma Acute Care Surg..

[19] Smith BK (2016). Diaphragm pacing as a rehabilitative tool for patients with pompe disease who are ventilator-dependent: Case series. Phys. Ther..

[20] Radunovic A, Annane D, Rafiq MK, Brassington R, Mustfa N (2017). Mechanical ventilation for amyotrophic lateral sclerosis/motor neuron disease. Cochrane Database Syst. Rev..

[21] Como JJ (2005). Characterizing the need for mechanical ventilation following cervical spinal cord injury with neurologic deficit. J. Trauma.

[22] Brown R, DiMarco AF, Hoit JD, Garshick E (2006). Respiratory dysfunction and management in spinal cord injury. Respir. Care.

[23] Supinski GS, Morris PE, Dhar S, Callahan LA (2018). Diaphragm dysfunction in critical illness. Chest.

[24] Chiuchiolo MJ (2014). Phase I/II study of intrapleural administration of a serotype rh.10 replication-deficient adeno-associated virus gene transfer vector expressing the human alpha1-antitrypsin cDNA to individuals with alpha1-antitrypsin deficiency. Hum. Gene Ther. Clin. Dev..

[25] Lin JY, Lin MZ, Steinbach P, Tsien RY (2009). Characterization of engineered channelrhodopsin variants with improved properties and kinetics. Biophys. J..

[26] Sunshine MD, Ganji CN, Reier PJ, Fuller DD, Moritz CT (2018). Intraspinal microstimulation for respiratory muscle activation. Exp. Neurol..

[27] Streeter KA (2019). Mid-cervical interneuron networks following high cervical spinal cord injury. Respir. Physiol. Neurobiol..

[28] Pickering M, Jones JF (2002). The diaphragm: Two physiological muscles in one. J. Anat..

[29] Xue J, Titterington D (2011). Median-based image thresholding. Image Vis. Comput..

[30] DeVries KL, Goshgarian HG (1989). Spinal cord localization and characterization of the neurons which give rise to the accessory phrenic nerve in the adult rat. Exp. Neurol..

[31] Gottschall J, Gruber H (1977). The accessory phrenic nerve in the rat. Ana. Embryol. (Berl).

[32] Chen IW, Papagiakoumou E, Emiliani V (2018). Towards circuit optogenetics. Curr. Opin. Neurobiol..

[33] Alilain WJ (2008). Light-induced rescue of breathing after spinal cord injury. J. Neurosc.: The off. J. Soc. Neurosci..

[34] Vajtay TJ (2019). Optogenetic and transcriptomic interrogation of enhanced muscle function in the paralyzed mouse whisker pad. J. Neurophysiol..

[35] Hwang HC (1995). Gene therapy using adenovirus carrying the herpes simplex-thymidine kinase gene to treat in vivo models of human malignant mesothelioma and lung cancer. Am. J. Respir. Cell Mol. Biol..

[36] Kucharczuk JC (1995). Pleural-based mesothelioma in immune competent rats: a model to study adenoviral gene transfer. Ann. Thorac. Surg..

[37] Mae M, Crystal RG (2002). Gene transfer to the pleural mesothelium as a strategy to deliver proteins to the lung parenchyma. Hum. Gene Ther..

[38] De B (2004). Intrapleural administration of a serotype 5 adeno-associated virus coding for alpha1-antitrypsin mediates persistent, high lung and serum levels of alpha1-antitrypsin. Mol. Ther..

[39] Falk DJ (2013). Intrapleural administration of AAV9 improves neural and cardiorespiratory function in pompe disease. Mol. Ther..

[40] Stiles KM (2018). Intrapleural gene therapy for alpha-1 antitrypsin deficiency-related lung disease. Chron. Obstr. Pulm Dis..

[41] Smuder AJ, Falk DJ, Sollanek KJ, Nelson WB, Powers SK (2013). Delivery of recombinant adeno-associated virus vectors to rat diaphragm muscle via direct intramuscular injection. Hum. Gene Ther. Methods.

[42] Mantilla CB, Zhan WZ, Sieck GC (2009). Retrograde labeling of phrenic motoneurons by intrapleural injection. J. Neurosci. Methods.

[43] Mah C (2004). A new method for recombinant adeno-associated virus vector delivery to murine diaphragm. Mol. Ther..

[44] Mah CS (2010). Gel-mediated delivery of AAV1 vectors corrects ventilatory function in Pompe mice with established disease. Mol. Ther..

[45] Naso MF, Tomkowicz B, Perry WL, Strohl WR (2017). Adeno-associated virus (AAV) as a vector for gene therapy. BioDrugs.

[46] Agbandje-McKenna M, Kleinschmidt J (2011). AAV capsid structure and cell interactions. Methods Mol. Biol..

[47] Gundelach LA, Huser MA, Beutner D, Ruther P, Bruegmann T (2020). Towards the clinical translation of optogenetic skeletal muscle stimulation. Pflugers Arch..

[48] Hanlon KS (2019). Selection of an efficient AAV vector for robust CNS transgene expression. Mol. Ther. Methods Clin. Dev..

[49] Davidoff AM, Ng CY, Zhou J, Spence Y, Nathwani AC (2003). Sex significantly influences transduction of murine liver by recombinant adeno-associated viral vectors through an androgen-dependent pathway. Blood.

[50] Bostick B, Ghosh A, Yue Y, Long C, Duan D (2007). Systemic AAV-9 transduction in mice is influenced by animal age but not by the route of administration. Gene Ther..

[51] Polinski NK (2016). Impact of age and vector construct on striatal and nigral transgene expression. Mol. Ther. Methods Clin. Dev..

[52] Fogarty MJ, Mantilla CB, Sieck GC (2018). Breathing: Motor control of diaphragm muscle. Physiology (Bethesda).

[53] Riaz M (2015). Differential myofiber-type transduction preference of adeno-associated virus serotypes 6 and 9. Skelet Muscle.

[54] McCall AL (2019). Reduction of autophagic accumulation in pompe disease mouse model following gene therapy. Curr. Gene Ther..

[55] Rasowo *et al.* Development of novel muscle specific adeno-associated viral vector constructs for gene therapy of duchenne muscular dystrophy. *Eur. Sci. J.* **10** (2014).

[56] Louboutin JP, Wang L, Wilson JM (2005). Gene transfer into skeletal muscle using novel AAV serotypes. J. Gene Med..

[57] Berlowitz DJ, Tamplin J (2013). Respiratory muscle training for cervical spinal cord injury. Cochrane Database Syst. Rev..

[58] Pettenuzzo T, Fan E (2017). 2016 year in review: Mechanical ventilation. Respir. Care.

[59] Onders RP (2018). Long-term experience with diaphragm pacing for traumatic spinal cord injury: Early implantation should be considered. Surgery.

