# Supplementary material for: Optogenetic activation of the diaphragm
Source: Sci Rep. 2022 Apr 20;12:6503. doi: 10.1038/s41598-022-10240-w (PMC9021282; doi:10.1038/s41598-022-10240-w)
Supplement: Supplementary file 4 — Supplementary Figure legends. [file 41598_2022_10240_MOESM4_ESM.docx]

**Title:** Optogenetic activation of the diaphragm

**Authors**: Ethan S Benevides^1,2,3,4^, Michael D. Sunshine^2,3,4^, Sabhya Rana^2,3,4^, *****David D. Fuller^2,3,4^

**Affiliations:** ^1^Rehabilitation Science PhD Program, University of Florida; ^2^Department of Physical Therapy, University of Florida; ^3^Breathing Research and Therapeutics Center, University of Florida; ^4^McKnight Brain Institute, University of Florida *Corresponding author

**Corresponding Author email:** dfuller@phhp.ufl.edu

**Journal:** *Scientific Reports*

**Submission date:** March 30, 2022

**SUPPLEMENTAL FIGURES**

| **Supplemental Figure 1. Representative example showing light activation of the diaphragm following bilateral section of the phrenic nerves**. (**a**) The left most traces show examples of diaphragm EMG activity at baseline. The middle traces show that after unilateral phrenic nerve transection ipsilateral diaphragm EMG output is attenuated. (**b**) Area under the curve analysis of diaphragm EMG (n= 5) at baseline and after bilateral phrenicotomy. Rank sum test showed a significant difference between area under the curve at baseline compared to bilateral phrenicotomy (p = 0.008). |
| --- |

| **Supplemental Figure 2. Power spectral density of the endogenous and evoked diaphragm EMG bursts.** (**a**) Average power of evoked diaphragm EMG bursts during baseline, bilateral phrenicotomy, and various stimulus conditions in mice (n= 5) that received a unilateral intrapleural injection of AAV9-CAG-ChR2-mVenus. (**b**) Bar graphs show average power in three power bands of interest, 0 – 333 Hz, 334-667 Hz, and 668-1001 Hz. Black lines indicated significant differences between groups (Friedman Repeated Measure Analysis of Variance on Ranks; p < 0.001; Tukey’s post-hoc; p < 0.05). |
| --- |

| **Supplemental Figure 3. Photostimulation without channelrhodopsin-2 transduction does not evoke diaphragm activity.** Example traces of the photostimulation (blue) and diaphragm EMG (black) for a naïve animal (n= 2 total) (**a**) and an animals that received a unilateral intrapleural injection of AAV9-CAG-ChR2-mVenus (**b**). (**c**) Stimulus triggered averages of diaphragm EMG activity verifies that in the absence of ChR2 there is no activation of the diaphragm during photostimulation and when ChR2 is present in diaphragm myofibers photostimulation is sufficient to evoke EMG activity. |
| --- |
